# Sparse Multivariate Analysis Reveals Dissociable White Matter Networks for Cognitive and Motor Processing Speed

**DOI:** 10.3390/brainsci16050533

**Published:** 2026-05-19

**Authors:** Shahwar Yasir, Nzamukiza Fidele, Eduardo Martinez-Montes, Lidice Galan-Garcia, Cheng Luo, Maria Luisa Bringas Vega, Pedro A. Valdes-Sosa

**Affiliations:** 1 Clinical Hospital of Chengdu Brain Science Institute, University of Electronic Science and Technology of China, Chengdu 610054, China; shahwar.yasir@neuroinformatics-collaboratory.org (S.Y.); chengluo@uestc.edu.cn (C.L.); maria.bringas@neuroinformatics-collaboratory.org (M.L.B.V.); 2 China-Cuba Belt and Road Joint Laboratory on Neurotechnology and Brain-Apparatus Communication, University of Electronic Science and Technology of China, Chengdu 611731, China; eduardo.martinez@neuroinformatics-collaboratory.org (E.M.-M.); lidice.galan@neuroinformatics-collaboratory.org (L.G.-G.); 3 GuangDong Mecable Communication Fiber Optical Cable Co., Ltd., Dongguan 523689, China; nzamfidele@gmail.com; 4 Cuban Neurosciences Center, Havana 11300, Cuba

**Keywords:** fractional anisotropy, reaction time, intra-individual variability, EEG alpha peak, white matter, canonical correlation analysis, multimodal neuroimaging

## Abstract

**Highlights:**

**What are the main findings?**

• Two distinct white matter networks were identified: one related to complex cognitive processing and another linked to motor response consistency.
• EEG alpha peak frequency did not show a significant relationship with reaction time performance.

**What are the implications of the main findings?**

• Cognitive and motor aspects of processing speed rely on different brain systems rather than a single mechanism.
• Brain structure may be more informative than the main frequency of resting-state EEG oscillations for understanding individual differences in processing speed.

**Abstract:**

**Background:** Reaction time (RT) is a fundamental measure of information processing speed in cognitive neuroscience and is influenced by both structural and functional brain properties. While prior studies have independently linked white matter microstructure and EEG alpha oscillations to cognitive performance, their joint contribution to distinct aspects of RT remains unclear. This study aims to investigate whether multimodal data can dissociate neural systems underlying cognitive and motor components of processing speed. **Methods:** We analyzed diffusion tensor imaging, resting-state individual EEG alpha peak frequency (IAF), demographic variables, and behavioral RT measures from a GO/NO-GO paradigm in 24 healthy adults from the Cuban Human Brain Mapping Project. Behavioral metrics included the mean, standard deviation and skewness of reaction times for simple and complex tasks. Sparse multiple canonical correlation analysis was applied to identify multivariate associations across modalities. **Results:** Two significant latent dimensions were identified. The first dimension linked bilateral fronto-temporal association tracts (SLF, IFOF, UNC) with complex RT performance, reflecting higher-order cognitive processing. The second dimension associated motor and interhemispheric tracts (CGC, CST, ILF, forceps major and minor) with intra-individual asymmetric variability (skewness) across tasks, indicating a motor-execution consistency system. IAF did not significantly contribute to either dimension. Sex showed strong associations with both components. **Conclusions:** Distinct white matter networks were associated with separable cognitive and motor aspects of processing speed, while resting-state alpha frequency did not show stable contributions with behavioral variability in this sample. IAF showed minimal contribution within the identified sparse multivariate dimensions. These findings highlight the importance of multimodal and multivariate approaches for understanding and potentially disentangling complex brain–behavior relationships.

## 1. Introduction

Neural basis underlying performance in human reaction time tasks remains a central topic in cognitive neuroscience, with roots extending to the foundations of psychometry and differential psychology established by Sir Francis Galton in the 19th century. Since then, numerous factors affecting reaction time performance have been evaluated, with methodologies evolving to use reaction time in perceptual and motor tasks to infer the content, duration, and temporal sequencing of cognitive operations. The reaction time increases with the amount of information conveyed in the stimulus and the options to respond, and is the final expression of complex cognitive processes integrating several additive factors, including the training [1], which can be eliminated by using experimental tasks designed with two level of difficulty, where the first level corresponds to the training.

White matter pathways connecting cortical regions are thought to be associated with individual differences in reaction time performance. The Diffusion-Weighted Imaging (DWI) modality boosts the research in mapping white matter pathways (tractography) and measuring white matter tracts’ integrity using fractional anisotropy (FA), which reflects their efficiency in neural signal transmission. The relationship between speed of processing and white matter microstructure has been studied for many years using different methodologies to answer diverse experimental questions related to the neural basis underlying cognitive processes and the distinct factors influencing them, especially age, sex, or education, among others. For example, Stufflebeam et al. [2] investigated the relation between timing of neural activity (latency of peak visual responses in occipital cortex measured by MEG) and white matter microstructural integrity using fractional anisotropy in the entire brain in eight healthy young adults. Penke et al. [3] studied elder people used a measure of integrity of eight major white matter tracts to predict a general factor of information processing speed. This analysis was effective for testing the overall combined effect of both tracts’ integrity measures and diverse types of reaction time measures. With a similar approach, Kuznetsova et al. [4] studied white matter structure integrity and information processing in healthy elders, using multiple linear regression to test the relationship between a ‘general factor of speed’ calculated from several measures of information processing and fractional anisotropy across the white matter in the whole brain.

In this study, the main result showed association of general speed factors with white matter skeleton integrity rather than specific regions. Individual reaction time response was revealed to be associated with neuroanatomy connectivity and white matter maturation and integrity [5]. For instance, for tasks that require interhemispheric information transfer and selection of bimanual response, it was found that the reaction time is negatively correlated with white matter FA of genus and body of corpus callosum, whereas tasks that require complex cognitive processing such as working memory are positively correlated with FA of frontal white matter [4].

On the other hand, electrophysiology can provide information about the association of electrical activity of the brain with behavior, for example, the hypothesis that certain types of brain waves (EEG rhythms) reflect mental efficiency (performance in reaction time). In an important seminal study, Klimesch et al. [6] demonstrated the strong relation of the alpha rhythm and the speed of processing information. The alpha rhythm is thought to reflect thalamocortical and corticocortical connectivity, with peak alpha frequency potentially indexing the speed of neural communication [7,8]. Valdés-Hernández et al. [9] provided evidence that white matter architecture, particularly in thalamocortical radiations, correlates with EEG alpha rhythm characteristics, supporting the hypothesis that structural connectivity constrains the frequency of oscillatory activity.

However, the extent to which these oscillatory properties directly predict behavioral performance in complex tasks, beyond their structural determinants, remains unclear. Indeed, the relationship between resting-state alpha peak frequency and task-specific cognitive performance may be indirect, mediated by structural pathways rather than reflecting direct functional contributions to information processing speed [9]. Challenges and problems for identification of anatomical and functional brain connectivity effects on inter-individual performance in reaction time task response could be only solved by multimodality experimental techniques and multivariate analysis methodologies able to effectively handle many variables associated with reaction tasks that involved mental processing speed.

For that reason, in this work we use more than one modality (structural and functional variables) in the same analysis to look for concurrent relationships between EEG alpha peak and white matter architecture (as reflected in FA data) with the speed of processing (reaction time) in healthy subjects. Among electrophysiological measures, individual EEG alpha peak frequency was selected because it has been proposed as a stable marker of neural communication efficiency and structural-functional brain organization [6,9]. The present study addresses three specific questions: (1) Do distinct white matter systems differentially support simple versus complex information processing demands? (2) Does resting-state EEG alpha peak frequency contribute independently to explaining reaction time variance beyond structural connectivity? (3) Can multivariate multimodal analysis disentangle separable brain–behavior relationships that would be obscured by traditional univariate approaches? We hypothesized that different tracts would be associated with performance on simple and more complex tasks requiring decision-making and rule maintenance, reflected in the intra-individual variability of motor execution speed as measured by the reaction times. At the same time, we hypothesize that the EEG alpha peak frequency would show limited direct association with behavioral performance in these complex cognitive tasks, potentially because individual alpha peak frequency may be partially associated with structural connectivity in thalamocortical networks rather than directly determining task-specific processing speed.

By applying sparse multiple canonical correlation analysis to DTI-based white matter integrity, EEG alpha peak frequency, demographic variables, and detailed reaction time measures (including mean, variability, and distributional parameters), we aim to demonstrate the utility of advanced multivariate methods for revealing distinct neural systems underlying information processing speed.

## 2. Materials and Methods

### 2.1. Sample

Twenty-four healthy subjects including 14 females and 10 males. All are right-handers, age range from 20 to 43 years old with mean age of 29.54 and SD of 8.5. This a subsample of the more than 400 subjects who were recruited at the Cuban Human Brain Mapping Project (CHBMP) (see description of the CHBMP, [10]). In this project, subjects were randomly selected in the population of the municipality of La Lisa, Havana. This population is considered representative in terms of ethnic and gender distribution of the Cuban population [11]. The curated dataset of this project has been published in [12].

Participants were included in the study after providing signed informed signed consent, in accordance with the ethical standards of the Declaration of Helsinki (Experimentation, 1964), and the experimental protocols were approved by the Ethics Committee of the Cuban Neuroscience Center. Each subject underwent an interview and medical examination with Neurology and Psychiatry specialists to rule out any pathology of the nervous system, which could invalidate their participation in the study. Neurological examination was performed following the procedure described in guidelines published by the Department of Health and Human Services U.S. in 2003. Mini-International Psychiatric Interview was used for psychiatric evaluation [13].

To check the cognitive status and discard mental disorders or other handicaps as exclusion criteria, the intelligence quotient (IQ) was obtained for each subject. The IQ was calculated using the Spanish language version of the Wechsler Adult Intelligence III Scale (WAIS III) [14].

### 2.2. Stimuli and Paradigm

**Figure 1:**
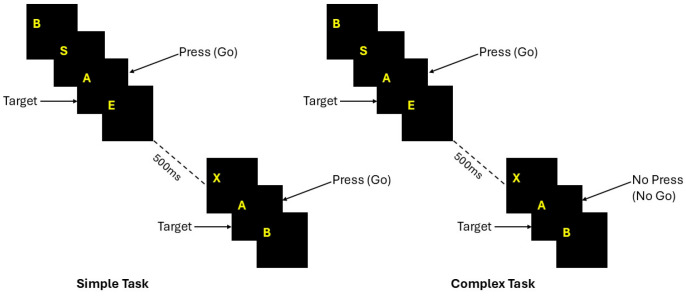
Experimental paradigm for simple and complex reaction time tasks. Both tasks required a motor response to target onset, but the complex task also required GO/NO-GO decisions based on stimulus context and task rules.

A visual GO/NO-GO task to record the reaction time was implemented using the software Mindtracer (Neuronic Cognitive Stimulator, version 2.1.0.1) for stimulation paradigms in psychophysiology, developed at the Cuban Neuroscience Center [15]. Each subject sat in front of a computer display and was instructed to press the ‘space’ key with the index finger of the right hand if the condition is correct. The duration of each stimulus wa 500 ms, and the screen cleared for 500 ms after a response was made, as shown in Figure 1.

The experimental paradigm consisted of two tasks, where a set of letters (P, B, X, E, A, S) were presented sequentially. Each task consisted of 500 trials and was applied with the following specific instructions as follows:

Simple Reaction Time (SRT): “Press the space bar when the letter “A” appears on the screen”.

Complex Reaction time (CRT): “Press the space bar only when the letter “A” appears preceded by the letter “S”.

The two tasks were presented to the subjects in the same consecutive order. Although this procedure might introduce a bias due to the order of the tasks, we were not interested in measuring the difference in reaction times, but the correlation between their values and the other biological or demographic variables. Instead, we believe that for this purpose it is adequate to have the same effect of training (if present) for all subjects. Schematic illustrations of the stimulus sequences used in the experiment are shown in Figure 1. In both tasks, participants fixate on a central stimulus showing a sequential presentation of black squares with a yellow uppercase letter in the center, and one of the letters is designated as the target. In the simple task (left), participants respond (press/GO) whenever the target appears, following a fixed delay (~500 ms). In the complex task (right), participants must additionally evaluate the stimulus context and selectively respond (GO) if the previous stimulus showed a specific letter or withhold response (NO-GO) if it did not, with 25% of the stimuli in GO condition and 75% in NO-GO condition. This design dissociates basic sensorimotor processing from higher-order cognitive control and decision-making processes [16].

For both simple and complex tasks, six behavioral variables were calculated for each subject, the mean of the response time for the trials with correct responses (reaction times), the standard deviation, and the skewness of the intra-subject distribution of reaction times, to study the influence of the asymmetry/variability of reaction times among subjects. Mean RT, RT variability (standard deviation), and RT distributional asymmetry (skewness) were treated as distinct behavioral metrics reflecting different statistical and potentially neurobiological aspects of performance. In addition, the mean and standard deviation of the errors (commission and omission errors for SRT and CRT) were also calculated for assessing that subjects have followed the instructions properly.

### 2.3. Acquisition and Preprocessing of Neuroimaging Data

Using a Siemens Symphony 1.5 T scanner (Erlangen, Germany), a 3D high-resolution T1 anatomical image and a standard low-resolution scheme of diffusion gradients were acquired for each subject. The T1 anatomical image was recorded with the following characteristics: 160 contiguous sagittal slices 1 mm thick, field of view (FOV) = 256 × 256 mm^2^, corresponding to a resolution in sagittal plane of 1 × 1 mm^2^, echo time (ET) = 3.93 ms, repetition time (RT) = 3000 ms. Using a single echo planar imaging (EPI) sequence, twelve diffusion-weighted images were obtained (b = 1200 s/mm^2^) and a reference T2 weighted image (b0 image) with no diffusion weighting (b = 0 s/mm^2^). The acquisition parameters were FOV= 256 × 256 mm^2^, acquired matrix = 128 × 128, corresponding to a resolution in the axial plane of 2 × 2 mm^2^, ET/RT= 160/7000 ms. The slice number was adapted to cover the whole brain with a slice thickness of 3 mm. The acquisition scheme was repeated 5 times to average the corresponding images and thus improving the signal-to-noise ratio. To correct the distortions caused by magnetic field inhomogeneities in the series of diffusion-weighted images, phase and magnitude maps were obtained. The parameters used were voxel size of 3.5 mm, ET1 = 7.71 ms, ET2 = 12.47 ms and RT = 672 ms.

All images were visually inspected, and those which presented either technical and/or pathological defects were discarded. Regarding the DWI images, a Hanning filter to b0 images was applied to correct the b0 diffusion images that presented a mild Gibbs ringing artifact around the ventricles. Other processing of this data was the correction of Eddy currents and motion effects by performing a linear registration of the weighted images to the b0, as well as the correction of distortions effects due to main field inhomogeneities by using the Unwarping SPM2 toolbox [17] on both phase and magnitude images. Diffusion tensors were fitted at every voxel using a linear regression method [18,19], and fractional anisotropy (FA) images were computed for all the subjects. To achieve anatomical correspondence, all individual FA images were normalized to the online template provided by the ICBM (ICBM-DTI-81) using SPM5 [20]. Since several factors, such as axonal density, myelination and axonal orientation homogeneity, influence the anisotropy value, a 3D anisotropic filter was applied to the warped FA images to reduce noise [21].

### 2.4. Estimation of the Diffusion Tensor and Fiber Tracking

Diffusion tensor and fiber tracking were estimated using the toolbox DTI & Fiber Tools v.3.0 [22], as described in detail in [11]. Briefly, three-dimensional reconstruction of white matter tracts was performed using the deterministic Fiber Assignment by Continuous Tracking (FACT) algorithm, following previously validated tractography protocols [23,24] using the Mori’s Atlas for ROI definitions [25]. Fiber tracking used predefined FA and angular stopping thresholds. Tract reconstruction was based on anatomically constrained multi-ROI selection procedures using established neuroanatomical landmarks. The ROIs were initially defined in standard MNI space and then automatically transformed into each subject’s native anatomical space. Deterministic tractography FACT was used to trace fiber orientation by starting on voxels corresponding to previously defined ROIs to estimate the tracts: anterior thalamic radiation (ATR), cingulate gyrus associated cingulum (CGC), hippocampal gyrus associated cingulum (CGH), cortico-spinal tract (CST), inferior fronto-occipital fasciculus (IFOF), inferior longitudinal fasciculus (ILF), superior longitudinal fasciculus (SLF), uncinate fasciculus (UNC), forceps major (Fmj) and forceps minor (Fmn). Reconstructed tracts were visually inspected and corrected, when necessary, through exclusion of anatomically implausible streamlines that did not conform to known tract anatomy. The mean, maximum, minimum, and standard deviation of FA values in voxels belonging to each tract are presented in Table 1.

### 2.5. EEG Data Recording and Preprocessing

EEG resting state was acquired using a 64 channel neurometric Cuban system (MEDICID-4, with software Neuronic EEG version 5.0.6.0) in an extended 10–20 montage, during 3 min of eyes closed condition. Detailed technical parameters can be found in prior methodological reports [9,26]. The EEG signal was band-pass filtered (0.5–45 Hz), down sampled to 100 Hz, and artifacts were removed using Independent Component Analysis (ICA). Power spectral density was computed using Fast Fourier Transform (FFT), and the alpha peak frequency was identified as the frequency with maximum amplitude between 7 and 13 Hz [6,27]. Spectral profiles and alpha peak estimations were visually inspected and verified by experienced neurophysiology experts as part of the quality-control procedures implemented within the Cuban Human Brain Mapping Project framework. Extremely noisy and uniformly distributed spectral profiles were excluded from analysis. The present study focused specifically on individual EEG alpha peak frequency (IAF) as the electrophysiological variable of interest because previous work has associated IAF with information processing speed, neural communication efficiency, and large-scale structural connectivity organization. In addition, limiting the EEG modality to a single scalar parameter reduced feature dimensionality and minimized overfitting risk given the modest sample size and the already high-dimensional multimodal dataset.

### 2.6. Research Design and Statistical Analysis

The final data to be analyzed consisted of three groups of variables: Group 1 consisted of 19 biological variables, namely the mean FA of eight tracts in each hemisphere and two interhemispheric tracts, and the EEG alpha peak; Group 2 contained 3 demographic variables, namely age, sex and educational level; Group 3 comprised the 6 behavioral variables, i.e., the mean, standard deviation and skewness of simple (SRT) and complex reaction times (CRT). Sex was the only not continuous variable, and it was coded as a binary demographic variable [0 = female, 1 = male]. Figure S1 of the Supplementary Material shows a correlation matrix among all variables.

To study the relationship between the FA in white matter tracts, the EEG alpha peak frequency and the speed of processing, we look for a unique model to explain these relations using canonical correlation (CCA) analysis, which has been traditionally used to identify and measure the associations among two sets of variables [28]. This approach has been extended to more than two datasets and is appropriate in the same situations where multiple regression would be, but when there are multiple intercorrelated outcome variables. Canonical correlation analysis determines a set of canonical variates (weights or loadings for each variable), which are orthogonal linear combinations of the variables within each set that best explain the variability both within and between sets.

On the other hand, the sample size was smaller (*n* = 24) than the number of variables (nv = 28); thus, it was necessary to apply regularization methods. For this purpose, we selected the sparse multiple canonical correlation analysis (sparse mCCA), which is a multivariate statistical analysis to test the strength of linear association between multiple sets of variables by maximizing the correlation between linear combinations of variables in each set [29].

We used the penalized matrix decomposition approach implemented in the PMA ‘R’ package (version 1.2-4) [30,31], and applied the MultiCCA function to the three groups of datasets. Each dataset was organized with subjects as rows and variables as columns. Before sparse multiple CCA analysis, all variables, except sex, were standardized to zero mean and unit variance to ensure comparability across behavioral, demographic, EEG, and white matter measures. The tuning parameters controlling sparsity were automatically estimated using the MultiCCA.permute procedure, which selects regularization parameters that maximize cross-dataset covariance (using 1000 permutations) while constraining the number of non-zero coefficients. Sparsity was imposed using an L1 (lasso) penalty, shrinking many coefficients exactly to zero and thereby improving interpretability by reducing the contribution of non-informative variables.

Statistical significance of the extracted canonical correlations was assessed using permutation testing within the PMA framework. A total of 5000 row-wise permutations were performed independently across groups to preserve within-group variance while destroying cross-group covariance structure. Empirical *p*-values were computed by comparing observed canonical correlations against the permutation-derived null distributions. We set *p* < 0.05 as the significance level for canonical correlations, corrected by Bonferroni to compare among the three pairwise correlations (corrected *p* < 0.017).

The same permutation test was used to assess the significance of the weights for each variable in each group. We used 5000 permutations, and the correction for multiple comparisons was performed using the maximum z-statistic approach [32]. Only the weighting parameters for the first and second sparse multiple CCA dimensions are reported, together with the highest corrected *p*-value for all significant variables in each group [30]. Although sparse regularization partially addresses the high dimensionality of the data, the stability of the extracted canonical weights was assessed by a leave-one-out procedure by subjects. This procedure provided a stability index representing the fraction of repetitions where each variable’s weight was nonzero. Nevertheless, given the relatively modest sample size, the identified canonical dimensions should be interpreted as exploratory multivariate association patterns. The analysis of the data and its interpretation was then oriented to address the following three questions: Which white matter tracts are significantly associated with behavioral measures? How strong and with which direction are the contributing variables in those associations? Which demographic variables have significant effects on FA and behavioral task performance? Is there any significant association between FA (white matter integrity), task performance, and brain functional efficiency measured by the frequency of the alpha peak?

## 3. Results

### 3.1. Behavioral Performance Characteristics

Table 2 summarizes behavioral performance across the SRT and CRT tasks, including mean reaction times, standard deviation, skewness, and number of errors. Overall, there were no significant differences between SRT and CRT responses, although the latter were slightly faster than the former on average. However, the standard deviation was higher for the CRT than for SRT, and the intra-subject distributions were positively skewed in both tasks, but stronger in the CRT. As expected, the CRT was also more error-prone than SRT, reflecting the greater task complexity. The total commission errors in the SRT tasks were 44, and omissions were 50. For the CRT task, total commission errors were 65, and omissions were 104. However, a chi-square test between them showed no statistical differences (F = 1.12; *p* = 0.28).

### 3.2. Canonical Correlations Among Groups of Variables

The inputs to the MultiCCA function were the three group datasets totaling 28 variables with 24 observations (subjects). The sparse mCCA analysis provided the canonical weights for each variable in the biological, demographic and behavioral groups, as well as the pairwise canonical correlations among these groups. Only two canonical dimensions were extracted because the average squared pairwise correlations between groups reached 52.6% of cumulative shared variance, with diminishing returns for a third dimension. Additionally, the leave-one-out stability analysis revealed that variables selected in a hypothetical third dimension had inclusion probabilities <0.15, indicating sample-specific noise. Given our sample size, retaining two sparse components optimizes the trade-off between model complexity and reproducibility. The corresponding optimal penalty values for biological, demographic and behavioral datasets were p1 = 3.15, p2 = 1.25, and p3 = 1.77, respectively, and were identical in both dimensions.

The canonical correlations between groups revealed moderate-to-strong multivariate associations across the extracted dimensions, whose significance was assessed by permutation testing corrected by Bonferroni (corrected *p* < 0.017). The canonical correlations between the biological and demographic groups were significant for both Dimension 1 (r = 0.67) and Dimension 2 (r = 0.59). Correlations between biological and behavioral groups showed weaker but significant values for both Dimension 1 (r = 0.55) and Dimension 2 (r = 0.56), whereas demographic–behavioral correlations were not significant for both dimensions (r = 0.37 and r = 0.19, respectively). The histograms of empirical null distributions for each pair of groups and each dimension are shown in Supplementary Material (see Supplementary Figure S2).

The next sections will detail results for the associated variables in each group according to their individual canonical weights or loadings. Because canonical weights in multivariate analyses reflect shared covariance structure across datasets, the estimated related contributions from each variable should not be interpreted as isolated effects on a single modality. Rather, the canonical weights likely reflect combined associations among behavioral performance, white matter microstructure, and their joint covariance patterns within the extracted latent dimensions.

### 3.3. Sparse Multiple CCA Identifies Two Significant Dimensions

Sparse canonical variate weights for the biological variables representing mean FA values, ranging from −1 to 1, are given for the two dimensions in Table 3. Higher weights imply higher contribution to the latent construct, which has maximal correlation among the three groups of variables. Weights that are zero can be interpreted as those variables that do not contribute to the correlation among the groups, i.e., do not show significant correlation with other variables. Non-zero values represent significant weights as assessed by the permutation test (corrected) for each dimension (the highest *p*-values were 0.015 and 0.013 for Dimension 1 and 2, respectively). The stability leave-one-out analysis demonstrated highly consistent loading patterns across iterations (mean loading correlation = 0.976), supporting the relative stability and robustness of the identified sparse multivariate dimensions despite the modest sample size.

**Figure 2:**
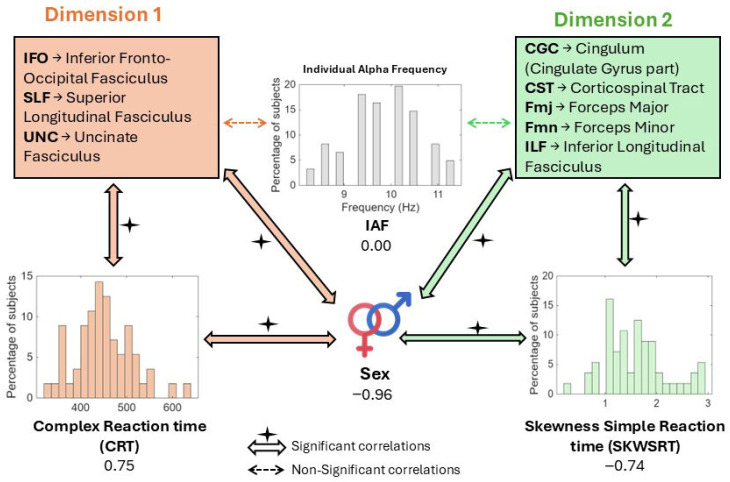
Conceptual summary of the dominant sparse mCCA latent loading patterns for significant weights. Dimension 1 (orange) involved association tracts (IFOF, SLF, UNC) linked with complex reaction time measures, whereas Dimension 2 (green) involved motor/interhemispheric tracts (CGC, CST, Fmj, Fmn, ILF) associated with reaction time skewness.

Figure 2 illustrates the global results for the two latent significant dimensions derived from the multimodal analysis. Dimension 1 is primarily associated with fronto-temporal white matter tracts, including the inferior fronto-occipital fasciculus (IFOF), superior longitudinal fasciculus (SLF), and uncinate fasciculus (UNC). These variables are positively related to the mean complex reaction time (CRT = 0.745) but also negatively with the asymmetry of this reaction time’s distribution (SKWCRT = −0.584). Dimension 2 primarily involves motor and interhemispheric pathways, including the cingulum (CGC), corticospinal tract (CST), forceps major (Fmj), forceps minor (Fmn), and inferior longitudinal fasciculus (ILF). This dimension is not significantly related to any of the mean reaction times, but it is negatively linked to skewness in both simple and complex reaction time measures (SKWSRT = −0.738; SKWCRT = −0.589). Both dimensions show a strong negative contribution from sex, while the individual alpha frequency (IAF) does not show a significant contribution in any of them.

### 3.4. Dimension 1: Fronto-Temporal Association Tracts and Complex Task Performance

**Figure 3:**
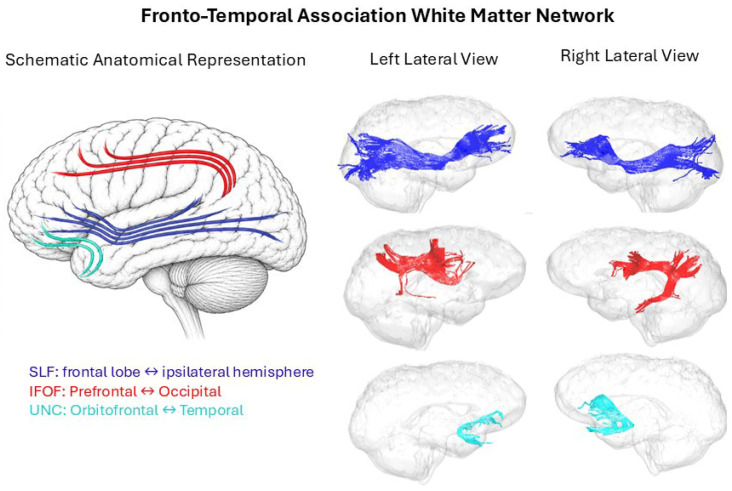
Schematic anatomical representation and glass-brain visualization of white matter tracts contributing to the multivariate association pattern related to complex reaction time performance. Shown are the superior longitudinal fasciculus (SLF), inferior fronto-occipital fasciculus (IFOF), and uncinate fasciculus (UNC) with left and right lateral tractography views.

The first dimension of sparse multiple CCA revealed significant weights for the white-matter mean FA of three bilateral tracts, namely the SLF, UNC and IFOF in both right and left hemispheres, as shown in Figure 3. The mean FA values for these tracts showed a significant association with the complex reaction time task, positively with the mean (CRT) and negatively with the skewness of CRT (SKWCRT). The complex reaction time task likely involved higher-order executive and attentional demands, including rule maintenance, response selection, language and cognitive control processes, associated with loading patterns involving SLF, IFOF, and UNC pathways. Interestingly, this appears only for the reaction times in the complex but not the simple task, suggesting that the simple task can be performed without involving the above-mentioned cognitive processes. At the same time, these processes seem to convey a more symmetric distribution of the complex reaction times, given the negative correlation with the skewness, which is high in this sample (see Table 2).

On the other hand, we found a strong negative relationship of the variable sex, reflecting an association with differences in the mean FA of significant tracts and/or in the performance of the complex task. In our dataset, we found that indeed females showed slower reaction times than males in both tasks, and they had also smaller mean FA in all tracts, which explains the negative association (see Table S1 and Figure S1 in Supplementary Material). Since the complex task may involve greater executive and attentional demands before performing a response on the subject, we can also identify this dimension as reflecting the ‘Complexity of the task’.

### 3.5. Dimension 2: Motor-Interhemispheric Tracts and Intra-Individual Variability

**Figure 4:**
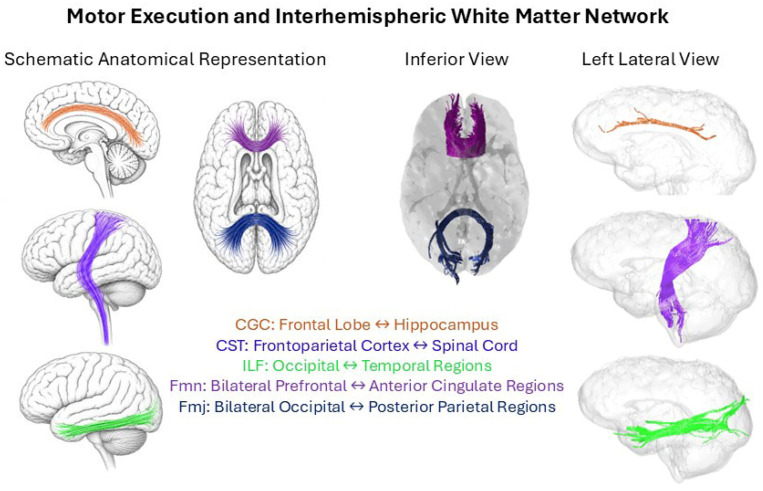
Schematic anatomical representation and glass-brain visualization of white matter tracts contributing to the motor execution and interhemispheric network. Shown are the cingulum bundle (CGC), corticospinal tract (CST), inferior longitudinal fasciculus (ILF), and the interhemispheric callosal fibers forceps major and minor (Fmj, Fmn).

The second dimension of the canonical correlation shows association between three bilateral and two forceps white matter tracts with intra-subject asymmetry of reaction times in both simple and complex tasks, as reflected by the skewness (SKWSRT and SKWCRT). The tracts are the CGC, CST and ILF in both hemispheres, plus the Fmj and Fmn, as shown in Figure 4. This dimension was associated with behavioral measures shared across both tasks, but it is not significantly related to the mean SRT or CRT, suggesting that differences in reaction times are more influenced by other networks and not by motor-related ones. The negative association with the skewness of both tasks’ reaction times suggests that higher mean FA in these tracts was associated with lower asymmetric variability of reaction times, i.e., making them less biased toward high values (even if they do not change in average). Thus, this dimension may reflect a multivariate association pattern related to intra-subject asymmetric variability. Importantly, the behavioral measures used in this study capture partially distinct aspects of processing speed. Mean reaction time reflects average response latency, standard deviation indexes intra-individual variability in response consistency, and skewness reflects asymmetry of the RT distribution, typically characterized by infrequent but disproportionately slow responses. Previous studies have shown that intra-individual variability in reaction time is associated with white matter maturation and neural efficiency [5]. In our study the metric with significant correlation was the skewness, which reflects the higher asymmetry towards slow responses. Therefore, these metrics should not be considered interchangeable or direct measures of processing speed, but they might both be complementarily reflecting distinct cognitive and motor processes. Like Dimension 1, the sex variable has also a strong negative contribution to the correlation among datasets, indicating that females are also showing higher mean FA values in the tracts that are significant in this canonical dimension (see Table S1 and Figure S1 in Supplementary Material).

### 3.6. Absence of Significant Associations for EEG Alpha Peak and Visual Pathways

Notably, the EEG alpha peak frequency did not show significant loadings on any of the canonical dimensions (Table 3 and Figure 2). Despite being included as a biological variable alongside white matter FA measures, alpha peak frequency was effectively excluded from both solutions by the sparsity constraints, indicating no substantial covariance with either the behavioral performance measures or the demographic variables in this sample (see Figure S1 in Supplementary Material).

Similarly, several white matter tracts associated with visual and medial temporal processing showed negligible contributions within the current sparse mCCA solution. The anterior thalamic radiation (ATR-L, ATR-R) and hippocampal cingulum (CGH-L, CGH-R) did not show stable non-zero loadings in either dimension. However, zero loadings in sparse multivariate models should not be interpreted as definitive evidence of functional non-involvement, since regularization procedures, shared variance between predictors, multicollinearity, and limited statistical power may reduce weaker contributions toward zero within the current model configuration. The absence of these visual and thalamocortical tracts from the significant weights, combined with the null finding for EEG alpha peak frequency, suggests that the observed brain–behavior relationships are specific to motor and association pathways rather than reflecting general visual processing speed or thalamocortical oscillatory properties.

## 4. Discussion

Our results identified distinct multivariate association patterns linking white matter organization with different aspects of information processing speed. We identified two significant latent dimensions: one linking fronto-temporal association tracts to complex task performance, and another linking motor-interhemispheric tracts to intra-individual asymmetric variability. Notably, resting-state EEG alpha peak frequency did not contribute significantly to either dimension, evidencing the lack of correlations between EEG alpha peak frequency and the other demographic and behavioral variables in this sample (see Figure S1 in Supplementary Material). These findings are consistent with our three hypotheses while highlighting the importance of multivariate approaches for disentangling complex brain–behavior relationships.

In the present study, the identified ‘fronto-temporal association’ and ‘motor execution’ dimensions should be interpreted as patterns of multivariate association emerging from the sparse mCCA framework rather than definitive or fully dissociable neurobiological systems. Although the observed loading profiles suggest partially distinct relationships between white matter integrity, EEG dynamics, demographic variables, and behavioral performance, the exploratory nature of the analysis and the relatively modest sample size limit strong conclusions regarding functional segregation. Therefore, these latent dimensions are better understood as preliminary multivariate patterns that may reflect partially overlapping neural processes requiring further validation in larger independent cohorts.

### 4.1. Distinct White Matter Systems for Cognitive Complexity and Motor Consistency

The first canonical dimension revealed a specific association between bilateral fronto-temporal white matter tracts—superior longitudinal fasciculus (SLF), inferior fronto-occipital fasciculus (IFOF), and uncinate fasciculus (UNC)—and performance on the complex reaction time task. This finding aligns with the established role of these association tracts in language and executive function. The SLF provides the anatomical substrate for transferring language information from posterior temporal regions to anterior frontal speech areas [33], while the IFOF has been implicated in semantic processing networks [34]. The UNC connects limbic structures (hippocampus, amygdala) with the orbitofrontal cortex and has been linked to language lateralization [35].

Our interpretation is that the CRT task, despite its apparent simplicity, imposes substantial cognitive demands through its verbal instructions and working memory requirements. Participants must maintain the conditional rule (‘S only if preceded by A’) while monitoring the stimulus stream, processes that engage in language comprehension and executive control systems supported by these tracts. The negative loading of skewness (SKWCRT) on this dimension suggests that individuals with higher FA in these tracts also show more consistent (less skewed) response distributions, indicating stable cognitive processing. This interpretation agrees with the ‘neural noise’ hypothesis, wherein better structural connectivity reduces variability in neural signal transmission [5,36].

The second dimension revealed a distinct anatomical system comprising the cingulate gyrus cingulum (CGC), corticospinal tract (CST), inferior longitudinal fasciculus (ILF), and both forceps tracts (Fmj, Fmn) associated with skewness measures across both simple and complex tasks. This pattern suggests a domain-general system supporting motor execution consistency and interhemispheric coordination. The CST provides the primary motor output pathway from cortex to spinal cord [37], while the CGC is implicated in motor control, conflict monitoring, and response inhibition [38]. The forceps tracts connecting occipital lobes via the splenium are associated with interhemispheric visual–motor integration [5], and the ILF conveys information between occipital and temporal lobes relevant for visual stimulus evaluation [39].

Importantly, this second dimension was associated with intra-individual asymmetric variability (skewness) across both task types, rather than mean response times. This finding converges with recent evidence that white matter microstructure in motor pathways specifically relates to response consistency rather than average speed. Hennessee et al. [40] demonstrated that neurite density in the superior CST correlated with non-decision time parameters in simple RT tasks, but not with mean RT itself, suggesting that motor pathway integrity primarily affects the efficiency of action initiation rather than decision processes. Our results extend this framework by showing that a broader network including cingulum and interhemispheric connections might support consistent motor execution across varying cognitive demands.

The distinction between these two dimensions suggests partially distinct multivariate association patterns related to cognitive complexity and motor consistency during information processing. This model posits that distinct neural circuits are linked with (1) the cognitive operations required for decision-making under complexity and (2) the efficient and consistent execution of motor responses. Our multivariate approach was essential for revealing this dissociation, as univariate analyses would be confounded by the substantial correlations between mean RT and variability measures, as well as between different white matter tracts. Importantly, the present sparse mCCA framework identifies latent multivariate covariance patterns rather than fully independent or biologically dissociable neural systems. Therefore, the identified dimensions should not be interpreted as evidence of strict functional segregation. Instead, the results suggest partially distinct association patterns linking different tract groups with behavioral measures within the current multivariate model. Considerable overlap and interaction between these white matter pathways likely exists, and the observed dimensions may reflect differential contributions rather thanseparate neural mechanisms.

Previous studies have demonstrated that white matter integrity is strongly associated with processing speed in healthy older adults [3,4], while increased reaction time variability has also been linked to age-related decline in white matter organization [5,36]. In traumatic brain injury (TBI), diffuse white matter disruption has been associated with impairments in cognitive speed, executive function, and behavioral consistency [41]. Importantly, several of the tracts identified in the present study, particularly the SLF, CST, cingulum, and interhemispheric forceps fibers, are among the pathways frequently reported as vulnerable in aging and TBI-related pathology [42,43]. Although few previous studies have specifically dissociated cognitive-complexity and motor-consistency systems using a multimodal multivariate framework, recent work has emphasized that distributed brain–behavior relationships are better captured using multivariate neuroimaging approaches rather than isolated univariate analyses [44]. These findings therefore suggest that the networks identified here may have broader relevance across neurological populations with altered structural connectivity. Future longitudinal and clinical studies using larger cohorts will be necessary to establish the robustness and translational relevance of these multimodal brain–behavior relationships.

### 4.2. Absence of EEG Alpha Peak Contributions: Structural Constraints on Oscillatory Activity

Our third hypothesis predicted limited direct association between EEG alpha peak frequency and behavioral performance, and this was confirmed: alpha peak frequency showed negligible loadings on both canonical dimensions. This null finding requires careful interpretation considering the extensive literature linking alpha oscillations to cognitive processing speed.

In the present study, we specifically examined the individual alpha peak frequency (IAF), rather than alpha oscillatory activity or alpha power more generally. Since IAF has been proposed to reflect conduction efficiency and structural organization within thalamocortical and corticocortical networks [6,9], it was included as a functional variable potentially related to processing speed. However, the absence of significant associations in our results should not be interpreted as evidence that structural connectivity is categorically more informative than EEG-derived measures for behavioral performance. Several methodological factors may have limited the sensitivity of the EEG analysis, including the use of resting-state alpha peak frequency as the sole electrophysiological metric, the relatively short EEG recording duration, and the modest sample size.

Importantly, previous studies have reported associations between other electrophysiological alpha measures, particularly alpha power, alpha reactivity, and task-related alpha modulation, with attention and reaction time performance [6,27]. Therefore, the present findings may indicate either that the alpha-related structural network is not directly involved in the specific processing demands of the reaction time tasks used here, or that the white matter tracts associated with reaction time performance are not the principal determinants of alpha peak frequency. Previous work has additionally shown that alpha rhythm properties are partially constrained by structural white matter organization, particularly thalamocortical pathways [9], supporting the interpretation that electrophysiological and structural measures may capture complementary aspects of neural organization. Future studies combining structural connectivity with task-related EEG dynamics and functional connectivity measures may therefore help clarify how distinct anatomical and electrophysiological mechanisms contribute to information processing speed.

Our findings are consistent with this structural-determinism view. The absence of alpha peak frequency contributions to the canonical dimensions suggests that, in our sample, individual differences in alpha frequency were either (1) insufficiently variable to contribute to behavioral predictions, or (2) redundant with the white matter measures already capturing individual differences in structural connectivity. The latter interpretation aligns with Valdés-Hernández et al. [9], finding that white matter architecture accounts for variance in alpha rhythms. In either case, our results indicate that for complex reaction time tasks involving language processing and motor execution, structural connectivity measures provide more direct and powerful prediction of individual differences than resting-state oscillatory markers.

This finding has methodological implications for studies seeking biomarkers of cognitive performance. While EEG offers practical advantages for large-scale screening, our results suggest that DTI-based structural measures may be more informative for understanding individual differences in task-specific processing speed. Nevertheless, future work should also study other measures related to EEG amplitude and employ simultaneous EEG-fMRI or combined EEG-DTI acquisition during task performance to analyze whether task-related (rather than resting-state) oscillatory dynamics show stronger behavioral associations.

### 4.3. Sex Differences in Brain–Behavior Relationships

Sex showed the strongest canonical weights (−0.96) across both dimensions and contributed substantially to the extracted multivariate association patterns. Although sex showed relatively strong loadings in the sparse mCCA dimensions, these findings should be interpreted cautiously given the modest and moderately unbalanced sample size. Previous studies using similar CHBMP diffusion imaging protocols have reported distributed sex-related differences across multiple white matter tracts rather than highly selective tract-specific effects [13,45,46]. The supplementary descriptive statistics revealed generally higher FA values across multiple tracts in females compared with males, consistent with previous CHBMP diffusion imaging findings [11] (see Table S1 in Supplementary Material). Similarly, in our sample, females also showed higher mean RT than males in both tasks. This finding is consistent with well-documented sex differences in reaction time performance, where males typically show faster responses, particularly for choice reaction time tasks [47]. Therefore, sex-related covariance may partially contribute to the observed multivariate structure. Although the latent dimensions identified in the present study were characterized by specific covariance patterns involving all modalities (behavioral measures, tract FA values, and EEG features), larger and more balanced cohorts will be necessary to more definitively disentangle sex-related and behavior-related contributions to the observed associations.

Recent large-scale studies have provided more nuanced perspectives on sex differences in white matter microstructure. Ingalhalikar et al. [48] analyzed 1062 young adults from the Human Connectome Project and found widespread higher fractional anisotropy in females across 40 out of 77 white matter tracts, with large effect sizes in the fornix and middle cortico-cerebellar tract. Similarly, Del Mauro et al. [49] found that women showed higher fiber density and fractional anisotropy in most tracts, particularly in the corpus callosum, fornix, and superior longitudinal fasciculus, even after adjusting for total brain volume. These microstructural advantages in females might be expected to confer processing speed benefits, yet our results and the broader literature show male advantages in RT performance.

This apparent paradox may reflect several factors. First, sex differences in RT may be primarily driven by non-structural factors such as motor execution efficiency, neuromodulatory tone, or strategy differences rather than white matter integrity per se. Second, the relationship between FA and processing speed is complex and tract-specific: higher FA in association tracts may support language processing (potentially advantageous in our verbal CRT task), while faster RT may depend more on CST microstructure where males may show advantages. Third, our sample size (*n* = 24) may have insufficient power to detect sex-by-tract interactions that would clarify these relationships. The ENIGMA consortium meta-analysis concluded that sex effects on white matter are modest (approximately 2% variance explained) and require large samples for robust detection [48,50].

### 4.4. Comparison with Existing Literature and Methodological Considerations

Our findings both converge with and extend previous research on white matter and reaction time. Tuch et al. [51] found that complex choice reaction time correlated with FA in visuospatial attention pathways (optic radiation, posterior thalamus, precuneus) but not motor pathways or corpus callosum, using voxel-based analysis. Our tract-specific approach revealed different associations—language tracts for CRT and motor/commissural tracts for variability—likely because our task employed verbal (letter) stimuli rather than visuospatial cues. This discrepancy highlights the importance of task-specific white matter associations: the neural substrates of processing speed are not universal but depend on the specific cognitive operations required.

The use of sparse multiple CCA represents a methodological advance over standard multiple regression approaches employed in previous studies [3,4]. While these studies demonstrate that white matter integrity predicts processing speed, they could not dissociate distinct systems for different aspects of performance. Our multivariate approach revealed the mean and skewness of the CRT load on different dimensions with different anatomical substrates, a finding that would be obscured in univariate analyses. This aligns with recent calls for multivariate methods in neuroimaging to capture the distributed nature of brain–behavior relationships [44].

The sparse CCA approach partially addresses the ‘curse of dimensionality’ inherent in multimodal studies by incorporating L1 regularization and variable selection. However, given the modest sample size, some instability of canonical weights cannot be excluded, and the extracted dimensions should therefore be interpreted cautiously until replicated in larger datasets.

### 4.5. Implications, Limitations, and Future Directions

Beyond the mechanistic interpretation of the identified white matter networks, these findings may also have important clinical implications. In the present work, intra-individual measures of reaction time variability, particularly skewness measures associated with motor and interhemispheric white matter pathways, were linked to differences in tract integrity across subjects. These findings suggest that assessing variability and asymmetry of responses in specific cognitive reaction time tasks could potentially be used in clinical settings to evaluate subtle alterations in structural connectivity in conditions such as sports-related concussion and traumatic brain injury. Importantly, variability measures may reveal impairments in response consistency even when average reaction times remain relatively preserved. This interpretation is consistent with prior studies showing associations between white matter integrity, reaction time variability, and cognitive stability [5,36]. Therefore, assessing reaction time distribution characteristics, rather than relying only on mean reaction time, may help support individualized diagnostic evaluation and the development of more targeted rehabilitation strategies.

Our findings suggest that interventions targeting processing speed deficits should consider specific cognitive operations impaired. For deficits in complex decision-making, enhancing integrity of fronto-temporal association tracts (SLF, IFOF, UNC) may be relevant, potentially through language-based cognitive training or neuromodulation approaches. For deficits in response consistency, targeting motor and interhemispheric pathways (CST, CGC, forceps) through motor skill training or physical exercise may be more appropriate.

Limitations of the present study should be acknowledged. First, the sample size (*n* = 24) is modest relative to the number of variables analyzed (nv = 28), and results require replication in larger cohorts. While sparse CCA incorporates regularization to handle high-dimensional data, the generalizability of canonical weights and the stability of dimension solutions would benefit from cross-validation in independent samples, beyond the leave-one-out analyses performed in this study. Therefore, the identified sparse mCCA dimensions should still be interpreted as exploratory multivariate association patterns requiring replication and validation in larger independent cohorts.

The fixed sequential presentation of SRT followed by CRT represents an important methodological limitation. Practice, expectancy, habituation, learning, or motor adaptation effects may have influenced mean reaction times, variability, skewness, and error distributions across tasks. This issue is particularly relevant when directly comparing behavioral performance between task conditions. Although the primary objective of the present study was to investigate multivariate associations between behavioral, structural, and electrophysiological measures rather than direct SRT-versus-CRT contrasts, sequential task effects may still have influenced the observed covariance patterns. At the same time, the use of a consistent task order across participants may have reduced additional inter-subject variability within the multivariate framework. Future studies employing randomized or counterbalanced task designs will be important for better dissociating task-complexity effects from sequential adaptation effects.

Additionally, the relatively low-resolution diffusion-weighted imaging protocol and the use of deterministic tractography (FACT algorithm) may have introduced measurement error in tract FA estimates. Several methodological limitations related to the diffusion imaging acquisition and tractography approach should be acknowledged. The diffusion-weighted imaging protocol employed a relatively low angular resolution together with deterministic FACT tractography, which may limit the accurate reconstruction of complex white matter architecture. In particular, deterministic streamline propagation is sensitive to crossing fibers, branching pathways, noise, and partial volume effects, potentially reducing anatomical precision in regions containing multiple fiber populations [24,51,52]. Consequently, the estimated FA values may reflect mixed microstructural contributions rather than tract-specific integrity alone, which may also influence the stability of the sparse mCCA associations.

Nevertheless, tract reconstruction followed previously validated ROI-based protocols developed by Mori and colleagues and later standardized by Wakana et al. [23,24,53], which were specifically designed to improve reproducibility and reduce variability in tract identification. Reconstructed tracts were additionally visually inspected and corrected, when necessary, through exclusion of anatomically implausible streamlines. Importantly, deterministic DTI tractography cannot fully resolve crossing fibers and may contain both false-positive and false-negative streamlines, particularly in peripheral white matter regions [24]. Although deterministic FACT tractography is relatively robust for reconstruction of major white matter bundles, it remains less accurate than probabilistic or multi-fiber approaches in regions containing complex fiber configurations. Therefore, the obtained FA estimates should be interpreted as approximations of large-scale white matter organization rather than precise representations of individual axonal architecture.

Additional limitations should also be considered. The use of resting-state EEG alpha peak frequency may not capture task-relevant oscillatory dynamics, and concurrent EEG acquisition during task performance could provide a more direct assessment of the relationship between electrophysiological activity and behavioral responses. Furthermore, the sparse CCA framework, while useful for variable selection and dimensionality reduction, assumes predominantly linear relationships and may therefore fail to capture potentially important non-linear brain–behavior associations. Sparse regularization procedures may also suppress weaker or highly correlated multivariate contributions, particularly in modest sample sizes. Finally, sex-related effects should be interpreted cautiously given the unequal sex distribution (14 females, 10 males) and the limited statistical power to evaluate sex-by-tract interaction effects.

Future work should address these limitations through several complementary directions. Larger samples with balanced sex distributions and counterbalanced task designs are needed to confirm the observed multivariate association patterns and clarify sex-specific brain–behavior relationships. Future diffusion imaging studies using higher angular resolution and multi-shell acquisitions, advanced diffusion models (e.g., NODDI and multi-compartment approaches), ultra-high field MRI (7T), and probabilistic or multi-fiber tractography methods may provide more accurate characterization of structural connectivity and improve reliability of multivariate brain–behavior associations. Simultaneous EEG-fMRI or combined EEG-DTI during task performance may further clarify whether task-related oscillatory dynamics show stronger behavioral associations than resting-state measures. Longitudinal studies tracking white matter organization and reaction time performance across the lifespan may help determine how these multivariate relationships evolve during maturation and aging. Additionally, although extensive EEG quality-control procedures were implemented, formal test–retest reliability of alpha peak estimation was not directly evaluated and should be addressed in future repeated-measure investigations. Finally, applying similar multimodal multivariate approaches to clinical populations with selective white matter pathology, such as multiple sclerosis, small vessel disease, or traumatic brain injury, may help establish the broader generalizability and clinical relevance of these brain–behavior associations.

## 5. Conclusions

This study identified multivariate association patterns linking white matter organization with different aspects of information processing speed using sparse multiple canonical correlation analysis of multimodal neuroimaging and behavioral data. Specifically, association tracts including the SLF, IFOF, and UNC were linked with behavioral measures derived from the complex reaction time task, whereas motor and interhemispheric pathways including the CGC, CST, ILF, and forceps tracts were associated with intra-individual variability measures across tasks. Resting-state EEG alpha peak frequency did not show significant contributions within the identified multivariate dimensions in this sample. Sex also contributed substantially to the observed covariance patterns.

These findings support the utility of multimodal multivariate approaches for studying distributed brain–behavior relationships that may not be fully captured by traditional univariate analyses. However, the identified dimensions should be interpreted cautiously given the modest sample size, exploratory design, and correlational multivariate framework. The present results do not establish causal or fully dissociable neural systems but rather suggest preliminary association patterns that require replication and validation in larger independent cohorts using stronger robustness and validation procedures.

Although structural neuroimaging measures showed stronger multivariate associations with behavioral performance than resting-state alpha peak frequency in the present dataset, electrophysiological and structural measures likely capture complementary aspects of neural organization. Future studies combining larger samples, longitudinal designs, advanced diffusion imaging, and task-related electrophysiological measures will be important for clarifying the stability, specificity, and generalizability of these multimodal brain–behavior relationships.

## Figures and Tables

**Table 1:** Summary of mean, range and standard deviation of FA values for the major white matter tracts and of the individual frequency of the alpha peak in the sample.

Neuroimaging Measures	Mean	Minimum	Maximum	Std. Dev.
ATR-L mean FA	0.445	0.331	0.601	0.087
ATR-R mean FA	0.480	0.365	0.654	0.087
CGC-L mean FA	0.417	0.305	0.517	0.067
CGC-R mean FA	0.404	0.319	0.530	0.060
CGH- L mean FA	0.355	0.228	0.456	0.082
CGH-R mean FA	0.335	0.219	0.491	0.081
CST-L mean FA	0.553	0.431	0.674	0.081
CST-R mean FA	0.586	0.386	0.747	0.107
Fmj mean FA	0.522	0.422	0.621	0.067
Fmn mean FA	0.457	0.367	0.575	0.074
IFOF-L mean FA	0.479	0.360	0.593	0.091
IFOF-R mean FA	0.447	0.352	0.531	0.069
ILF-L mean FA	0.467	0.346	0.607	0.090
ILF-R mean FA	0.464	0.351	0.792	0.105
SLF-L mean FA	0.465	0.336	0.596	0.091
SLF-R mean FA	0.463	0.338	0.621	0.089
UNC-L mean FA	0.379	0.257	0.487	0.080
UNC-R mean FA	0.369	0.275	0.481	0.073
Alpha Peak Frequency	10.303	8.594	11.328	0.718

**Table 2:** Summary of the simple and complex task performance variables (mean, std, skewness of reaction times, and errors) for the GO, NO-GO experiment.

Variable	Mean	Minimum	Maximum	Std. Dev
SRT	463.54	408.00	518.00	35.49
CRT	433.37	357.00	555.00	47.14
SDSTR	78.58	44.04	114.78	17.59
SDCRT	85.81	45.24	138.71	22.75
SKWSRT	1.08	−0.36	2.10	0.59
SKWCRT	1.41	−0.41	2.41	0.55
Commission errors SRT	1.80	0	9	2.10
Omission errors SRT	2.08	0	15	1.92
Commission errors CRT	2.71	0	12	2.56
Omission errors CRT	4.33	0	18	4.21

**Table 3:** Sparse canonical variate weights for the two significant dimensions.

Variables	Dimension 1 Canonical Variate Weights (*p* < 0.015)	Dimension 2 Canonical Variate Weights (*p* < 0.013)
ATR-L mean FA	0	0
ATR-R mean FA	0	0
CGC-L mean FA	0 *	0.363 *
CGC-R mean FA	0 *	0.303 *
CGH-L mean FA	0	0
CGH-R mean FA	0	0
CST-L mean FA	0 *	0.357 *
CST-R mean FA	0 *	0.346 *
Fmj mean FA	0	0.373 *
Fmn mean FA	0	0.307 *
IFOF-L mean FA	0.333 *	0 *
IFOF-L mean FA	0.337 *	0 *
ILF-L mean FA	0	0.301 *
ILF-R mean FA	0 *	0.409 *
SLF-L mean FA	0.423 *	0 *
SLF-R mean FA	0.439 *	0 *
UNC-L mean FA	0.379 *	0 *
UNC-R mean FA	0.345 *	0 *

Weights marked with an asterisk (*) correspond to variables showing a stability index > 0.75 for non-zero loadings and <0.25 for stable zero loadings.

## Data Availability

The dataset included BIDS files, the in-house programs, the psychological (WAIS-III, MMSE and reaction time), and the demographic and handedness data (∗.csv) available at https://www.synapse.org/. See reference [3]. You can visualize the data at https://doi.org/10.7303/syn22324937. To download them you need to be registered at the synapse.org website. All the datasets have also been stored in the McGill Centre for Integrative Neuroscience (MCIN) network. The dataset will be available by request at https://chbmp-open.loris.ca.

## Supplementary Materials

The following supporting information can be downloaded at https://www.mdpi.com/article/10.3390/brainsci16050533/s1, Figure S1: Correlation matrix of biological, demographic, and behavioral variables included in the sparse multivariate analysis; Figure S2: Permutation-derived null distributions of canonical correlations for the sparse mCCA dimensions; Table S1: Descriptive statistics stratified by sex for the main demographic, behavioral, EEG, and white matter variables included in the sparse mCCA analysis, including reaction time measures and tract FA values.

## Abbreviations

The following abbreviations are used in this manuscript:

ATR	Anterior Thalamic Radiation
CGC	Cingulate Gyrus Cingulum
CGH	Hippocampal Gyrus Cingulum
CST	Corticospinal Tract
DTI	Diffusion Tensor Imaging
DWI	Diffusion-Weighted Imaging
EEG	Electroencephalography
FA	Fractional Anisotropy
Fmj	Forceps Major
Fmn	Forceps Minor
FFT	Fast Fourier Transform
FOV	Field of View
GO/NO-GO	Go/No-Go Task Paradigm
IAF	Individual Alpha Frequency
ICA	Independent Component Analysis
IFOF	Inferior Fronto-Occipital Fasciculus
ILF	Inferior Longitudinal Fasciculus
mCCA	Multiple Canonical Correlation Analysis
PMA	Penalized Matrix Decomposition
RT	Reaction Time
SRT	Simple Reaction Time
CRT	Complex Reaction Time
SDSTR	Standard Deviation of Simple Reaction Time
SDCRT	Standard Deviation of Complex Reaction Time
SKWSRT	Skewness of Simple Reaction Time
SKWCRT	Skewness of Complex Reaction Time
SLF	Superior Longitudinal Fasciculus
UNC	Uncinate Fasciculus
