# Supplementary material for: Sparse Multivariate Analysis Reveals Dissociable White Matter Networks for Cognitive and Motor Processing Speed
Source: Brain Sci. 2026 May 19;16(5):533. doi: 10.3390/brainsci16050533 (PMC13204814; doi:10.3390/brainsci16050533)
Supplement: Supplementary file 1 [file brainsci-16-00533-s001.zip › brainsci-4281444-supplementary.pdf]

Supplementary Table S1: Descriptive statistics stratified by sex for the main demographic, behavioral, EEG, and white matter variables included in the sparse mCCA analysis, including reaction time measures and tract FA values.

	Males (N = 10)		Females (N = 14)		H0: Male >=Female	
	**Mean**	**Std**	**Mean**	**Std**	**T-stat**	**p-value**
**DEMOGRAPHIC**						
Sex	-	-	-	-	-	-
Schooling	12.300	1.767	12.429	2.766	-0.139	0.445
Age	26.100	4.654	32.000	6.300	-2.638	0.008
**BEHAVIORAL**						
RT						
SRT	459.900	34.904	466.143	36.982	-0.421	0.339
CRT	422.800	45.524	440.929	48.474	-0.936	0.180
SDSTR	77.210	15.543	79.561	19.438	-0.329	0.373
SDCRT	77.994	15.848	90.882	25.841	-1.510	0.073
SKWSRT	1.092	0.584	1.074	0.627	0.075	0.471
SKWCRT	1.573	0.211	1.309	0.696	1.338	0.100
**BIOLOGICAL**						
EEG						
Alpha Peak Freq	10.234	0.576	10.352	0.822	-0.411	0.343
Mean FA						
left ATR	0.384	0.062	0.489	0.076	-3.734	0.001
right ATR	0.424	0.073	0.520	0.074	-3.159	0.003
left CGC	0.379	0.064	0.445	0.056	-2.632	0.009
right CGC	0.371	0.057	0.427	0.052	-2.465	0.012
left CGH	0.300	0.074	0.395	0.064	-3.275	0.002
right CGH	0.280	0.082	0.373	0.055	-3.131	0.004
left CST	0.503	0.056	0.589	0.078	-3.127	0.002
right CST	0.531	0.088	0.626	0.105	-2.402	0.013
Fmj	0.474	0.052	0.557	0.055	-3.779	0.001
Fmn	0.410	0.064	0.491	0.062	-3.117	0.003
left IFOF	0.413	0.078	0.526	0.069	-3.685	<0.001
right IFOF	0.391	0.054	0.488	0.047	-4.585	0.000
left ILF	0.405	0.086	0.511	0.066	-3.260	0.002
right ILF	0.399	0.066	0.511	0.104	-3.237	0.002
left SLF	0.397	0.067	0.513	0.073	-4.028	<0.001
right SLF	0.396	0.065	0.511	0.072	-4.071	<0.001
left UNC	0.324	0.065	0.418	0.067	-3.479	0.001
right UNC	0.314	0.048	0.409	0.061	-4.282	<0.001

The following table reports descriptive statistics stratified by sex for the main demographic, behavioral, EEG, and white matter variables included in the sparse mCCA analysis. Behavioral measures include simple and complex reaction time (RT) mean, standard deviation (STD), and skewness (SKEW), while EEG measures include individual alpha peak frequency. Fractional anisotropy (FA) values are reported for the analyzed white matter tracts in male and female participants. T-statistics and p-values correspond to two-sample t-tests assuming unequal variances between groups. Consistent with previous CHBMP diffusion imaging findings, females showed generally higher FA values across multiple white matter tracts compared with males, whereas behavioral and EEG differences were comparatively smaller. These descriptive statistics provide additional context for interpreting the sex-related covariance patterns observed in the sparse mCCA dimensions. Abbreviations: ATR = anterior thalamic radiation; CGC = cingulum bundle; CGH = hippocampal cingulum; CST = corticospinal tract; Fmj = forceps major; Fmn = forceps minor; IFOF = inferior fronto-occipital fasciculus; ILF = inferior longitudinal fasciculus; SLF = superior longitudinal fasciculus; UNC = uncinate fasciculus.

Supplementary Figure S1: Correlation matrix of biological, demographic, and behavioral variables included in the sparse multivariate analysis.

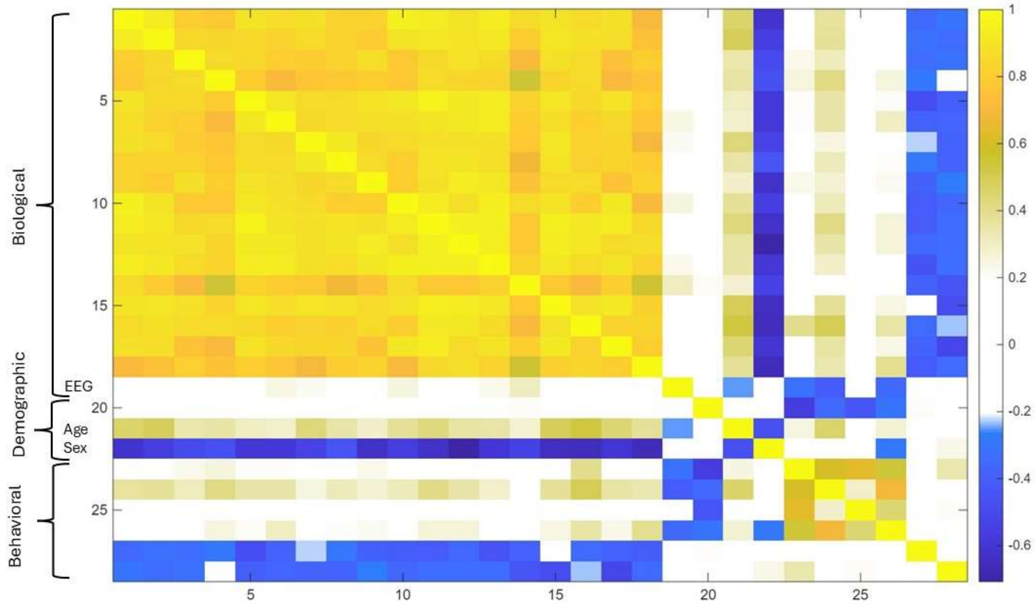

Heatmap showing pairwise Pearson correlation coefficients among white matter tract fractional anisotropy (FA) measures and EEG alpha peak frequency (biological variables), demographic variables (schooling, age and sex), and behavioral reaction time measures derived from the simple and complex tasks. Warmer colors indicate positive correlations, whereas cooler colors indicate negative correlations. Biological variables showed

predominantly moderate-to-strong positive intercorrelations, particularly among homologous white matter tracts, reflecting shared microstructural covariance patterns across the brain. Behavioral variables also demonstrated positive associations among reaction time measures and variability metrics. Sex showed widespread negative correlations with several tract FA and behavioral variables, consistent with group differences observed in the sample. In contrast, EEG alpha peak frequency demonstrated relatively weak correlations with both behavioral and structural variables. Overall, the matrix illustrates the covariance structure underlying the sparse multivariate canonical correlation analysis.

Supplementary Figure S2. Permutation-derived null distributions of canonical correlations for the sparse mCCA dimensions.

Histograms show empirical null distributions of canonical correlations obtained from 5000 permutation iterations independently applied across the biological (BIO), demographic (DEMO), and behavioral (BEHAV) blocks. Red vertical lines indicate the observed canonical correlations obtained from the original sparse mCCA solution. Left column corresponds to Dimension 1 and right column to Dimension 2. Top row shows BIO–DEMO correlations, middle row BIO–BEHAV correlations, and bottom row DEMO–BEHAV correlations.

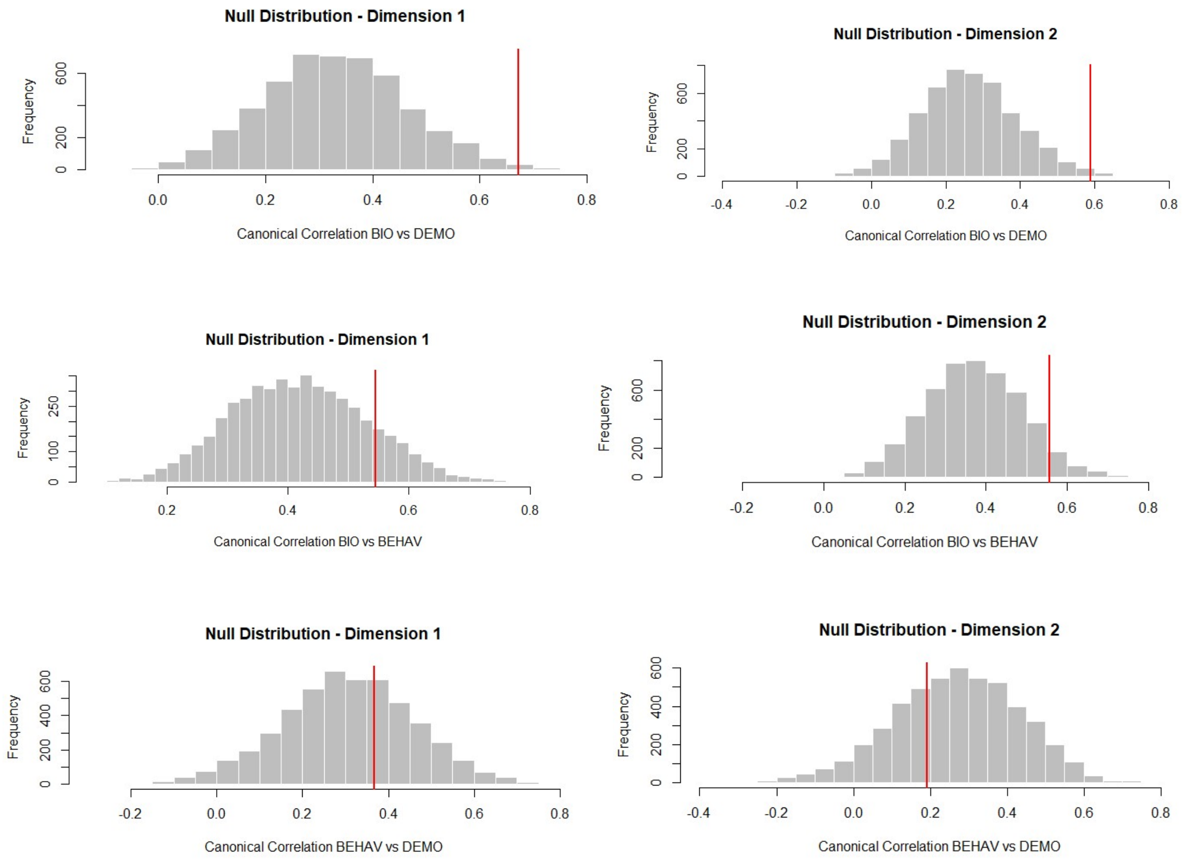

Permutation testing demonstrated that the observed canonical correlations between the biological and demographic blocks exceeded the empirical null distributions in both latent dimensions, supporting the presence of statistically meaningful multivariate covariance patterns between white matter organization and demographic variables. The biological–behavioral correlations also showed moderate effect sizes and were located toward the upper tail of the null distributions, particularly for Dimension 2, indicating stronger-than-chance multivariate associations between tract FA measures and reaction time variables. In contrast, the demographic–behavioral correlations showed weaker separation from the null distributions and did not reach statistical significance, suggesting comparatively weaker shared covariance between these blocks within the current sparse multivariate framework.

Overall, the permutation analysis supports the existence of stable multivariate association patterns linking structural connectivity, demographic factors, and behavioral performance while also illustrating differences in association strength across the extracted latent dimensions.
